# Large‐scale NMR simulations in liquid state: A tutorial

**DOI:** 10.1002/mrc.4660

**Published:** 2017-11-27

**Authors:** Ilya Kuprov

**Affiliations:** ^1^ School of Chemistry University of Southampton University Road Southampton SO17 1BJ UK

**Keywords:** NMR, simulation, spin, spinach, software

## Abstract

Liquid state nuclear magnetic resonance is the only class of magnetic resonance experiments for which the simulation problem is solved comprehensively for spin systems of any size. This paper contains a practical walkthrough for one of the many available simulation packages — Spinach. Its unique feature is polynomial complexity scaling: the ability to simulate large spin systems quantum mechanically and with accurate account of relaxation, diffusion, chemical processes, and hydrodynamics. This paper is a gentle introduction written with a PhD student in mind.

## 1 INTRODUCTION

Textbooks and introductory lectures make nuclear magnetic resonance (NMR) simulations look deceptively simple: type in some Pauli matrices, make a Hamiltonian, compute the exponential, and that's ostensibly it — their authors have done a wonderful job of making the subject easy to understand.^1^, ^2^, ^3^ The reality is rather more brutal: relaxation theory requires deep knowledge of tensor calculus, interaction specifications and rotation conventions are a veritable minefield, matrix manipulation is a highly technical subject… and then there are chemical kinetics, diffusion, flow, spatial encoding, distant dipolar effects, hyperpolarisation, and paramagnetic shifts. With a bit of luck, the simulation would be done by the end of the PhD project… or maybe not. Fortunately, there is now an app for that, and it is called *Spinach*.^4^

This paper is a practical walkthrough — it goes through the process of setting up and running liquid state NMR simulations in the order that most people would be doing it in practice. The purpose of *Spinach* in this context is to simplify the process: the program automates all intermediate stages (Hamiltonian generation, relaxation superoperator calculation, time evolution mathematics, *etc.*) and offers many standard pulse sequences as pre‐programmed modules with detailed examples and documentation. Complicated particulars of the internal mathematics and programming are avoided as much as possible here, with references to the more technical papers.

At the time of writing, *Spinach* is unique in its ability to simulate, without significant approximations 1
*Spinach* drops unpopulated quantum states — this reduces the basis set and makes calculations faster but does not influence accuracy of the final answer. Technical details are published in Karabanov et al.^28^
 and in the time domain, liquid state NMR systems containing hundreds of interacting spins.^5^ Many packages can generate a reasonable likeness of a 1D NMR spectrum for large spin systems, but complicated combinations of multidimensional pulse sequences, advanced relaxation and kinetics treatments, shaped pulses and gradients, diffusion, and flow are only available in *Spinach*. This is the result of very recent theoretical developments, the primary ones being quantum mechanical simulation algorithms^6^, ^7^ that have much lower computational resource requirements than anything previously available, and the Fokker–Planck equation for the spatial degrees of freedom.^8^, ^9^

Spinach is a *Matlab* package, the primary reason being convenience: of all available scientific computing environments, *Matlab* takes the shortest amount of time to get a calculation going. To set *Spinach* up, follow the installation instructions on the website (http://spindynamics.org). The current public version requires *Matlab R2016b* or later with *Parallel Computing Toolbox* and *Optimisation Toolbo*x installed.

## 2 WHAT DOES NMR SIMULATION SOFTWARE DO?

Time domain NMR simulation packages solve Liouville ‐ von Neumann's equation (the equivalent of Schrödinger's equation for spin ensembles) and calculate the observable magnetisation at each point in time^10^:

(1) $$\frac{\partial }{\partial t}\hat{\rho }\left(t\right)=-i\hat{\hat{L}}\left(t\right)\hat{\rho }\left(t\right)\;m\left(t\right)=\left\langle \hat{m}|\hat{\rho }\left(t\right)\right\rangle ,$$

where $\hat{\rho }\left(t\right)$ is a vector that contains information about spin system state, $\hat{\hat{L}}$ is a matrix, called Liouvillian, that depends on things such as *J*‐couplings and relaxation rates, and $\hat{m}$ is the observable magnetisation projector. To a computer, Equation (1) looks like standard linear algebra; it is solved by calculating the exponential of $\hat{\hat{L}}$:

(2) $$\hat{\rho }\left(t+\mathrm{dt}\right)=\exp\left[-i\hat{\hat{L}}\left(t\right)\mathrm{dt}\right]\hat{\rho }\left(t\right).$$

Technical details may be found in more specialised reviews of magnetic resonance simulation methods.^9^, ^10^, ^11^, ^12^, ^13^, ^14^ *Spinach* is designed to automate this process: the user specifies the spin system and the experiment parameters, and receives a free induction decay at the end of the calculation.

**Figure 1:**
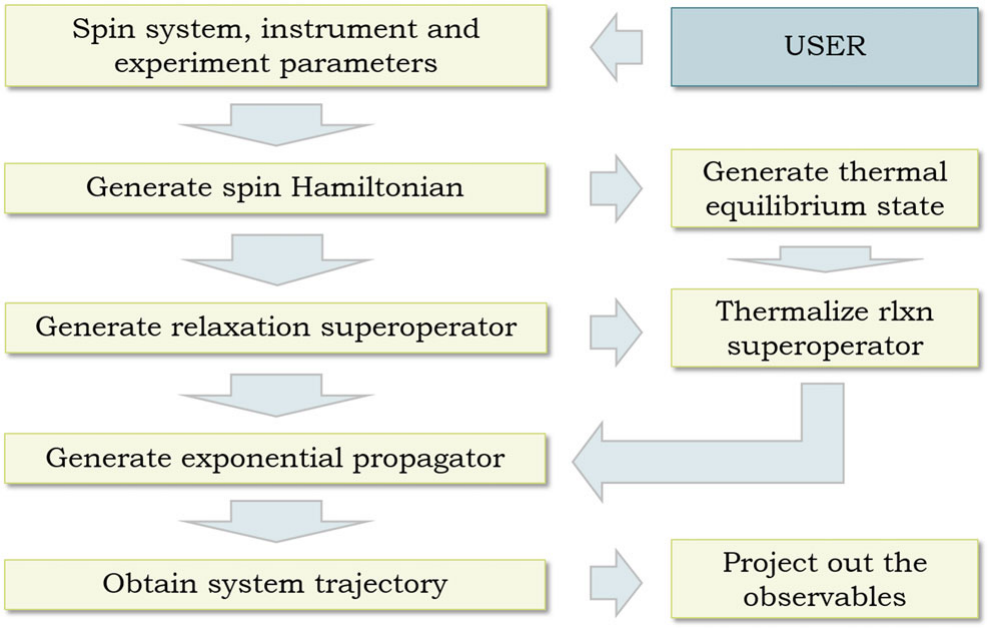
Time‐domain NMR simulation flowchart. All stages except the first are automated in modern magnetic resonance simulation software

Figure 1 shows the general flowchart of a typical liquid state NMR simulation. The job of the user is to say which interactions are active at which time, to specify the molecule, and to choose the pulse sequence. *Spinach* builds and solves Equation (1), and returns the answer to the user.

## 3 SPECIFYING THE SYSTEM

In order to be understood by a simulation package, spin system parameters (chemical shifts, *J*‐couplings, *etc.*) must be specified in a certain formal way that the program expects. Standard formats are starting to emerge,^15^ but at the moment, every simulation package has its own way of specifying the spin system. *Spinach* uses *Matlab* data structures that are described in this section.

Any *Spinach* calculation must begin with a specification of three major aspects of the simulation:

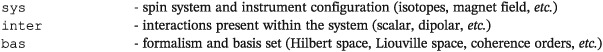

*Matlab* uses dots to separate fields in its data structures. Those fields make a convenient hierarchy that is used to supply information to *Spinach*, for example,

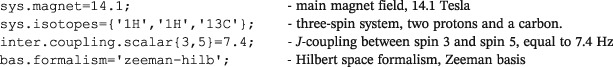

Statements of this kind are described in detail in the manual (http://spindynamics.org/wiki). Once the specification is typed in, the three data structures sys, inter, and bas must be supplied to create.m and basis.m constructor functions. These functions process spin system and simulation formalism specifications, write some useful diagnostics to *Matlab* console and create the spin_system object — the primary data structure that is used to store spin system information in *Spinach*:

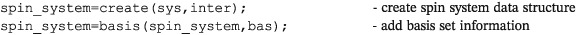

Once these functions are run, *Spinach* has all the necessary information about the spin system and the formalism. The program performs extensive input validation and will always tell the user if it needs more information. A typical specification for a simple liquid state NMR case looks like the following:

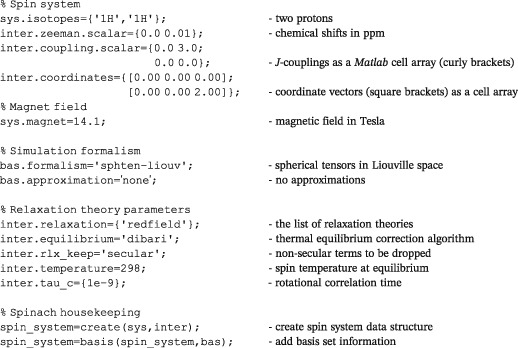

It is clear that the specification is human‐readable — a quick way to get going is to modify one of the many standard examples supplied with *Spinach*. *Matlab* has three types of brackets: round brackets are used for function arguments and array indices, square brackets are used for vectors and matrices, and curly brackets are used for arrays that can contain anything — those are called cell arrays. This latter type is needed for arrays with flexible structure, for example, rotational correlation times may be different for different chemical species, and each of those species may have a different number of them when rotational diffusion is anisotropic. Further details of the input syntax are given in the sections below. Deeper technicalities are in the online manual.

### 3.1 Isotopes and labels

Spin system composition is specified by giving a list of isotope names, for example,



sys.isotopes={'1H','1H','19F','235U'};



All known isotopes are supported, including those with spin zero. Optionally, a label for each spin may be specified by giving a list of strings, for example,



sys.labels={'CA','CB','HB2','HB3'};



Labels are printed next to spin interaction summaries — this makes diagnostic output easier to read for large spin systems. Labels are also used by protein NMR spectroscopy modules to identify different types of atoms — when a dedicated protein pulse sequence (such as hncoca.m) is run, these labels must be set to the standard Protein Data Bank (PDB) atom identifiers. PDB and Biological Magnetic Resonance Bank (BMRB) import functions set these labels automatically.

### 3.2 Interactions

There are three broad classes of interactions in NMR — between spins and the external magnetic field, between spins and other spins, and inside (or so it looks) a specific spin. Mathematically, all three classes have the same appearance — as a product of two spin vectors ${\vec{s}}_{1}$ and ${\vec{s}}_{2}$ with a matrix **A** in the middle:

**Table:** 

Interaction type	Mathematical form	Examples
Spin–field	${\vec{s}}_{1}^{T}\cdot A\cdot \vec{B}$	Chemical shift
Spin–spin	${\vec{s}}_{1}^{T}\cdot A\cdot {\vec{s}}_{2}$	Dipolar coupling
Internal	${\vec{s}}_{1}^{T}\cdot A\cdot {\vec{s}}_{1}$	Quadrupolar coupling

The matrix is called “interaction tensor”. Its orientation‐independent (“isotropic”) part is responsible for the line pattern in the NMR spectrum, and the part that changes with molecular orientation (“anisotropic”) is responsible for the line width and other relaxation properties.

For the spin–field interactions, *Spinach* needs the primary magnet field in units of Tesla, for example,



sys.magnet=14.1;



If the system has chemical shifts, they may be specified as scalars, 3 × 3 matrices, or eigenvalues + Euler angles (in radians). If multiple specifications are supplied, they are added together.

**Table:** 

Variable name	Variable type	Content
inter.zeeman.eigs inter.zeeman.euler	[1 × nspins] cell array of [1 × 3] row vectors	Eigenvalues of chemical shift tensors (in ppm) with Euler angles (in radians).
inter.zeeman.matrix	[1 × nspins] cell array of [3 × 3] matrices	Full chemical shift tensors (in ppm) as matrices.
inter.zeeman.scalar	[1 × nspins] cell array of real numbers	Isotropic chemical shifts (in ppm).

Examples:

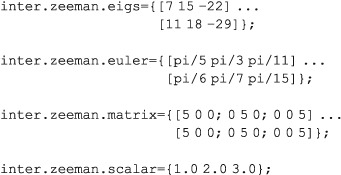

Spin–spin couplings may also be specified as scalars, 3 × 3 matrices, or eigenvalues + Euler angles. If multiple specifications are supplied, *Spinach* adds them together.

**Table:** 

Variable name	Variable type	Content
inter.coupling.eigs inter.coupling.euler	[nspins × nspins] cell array of [1 × 3] matrices	Eigenvalues of coupling tensors (in Hz) with Euler angles (in radians). Bilinear coupling is introduced by specifying a coupling between two spins. Quadratic coupling (*e.g.* quadrupolar) is introduced by specifying a coupling between a spin and itself.
inter.coupling.matrix	[nspins × nspins] cell array of [3 × 3] matrices	Full coupling tensors as matrices (in Hz). Each element of the cell array is accounted for, so the couplings must be divided by two if a symmetric cell array is supplied.
inter.coupling.scalar	[nspins × nspins] cell array of reals	Isotropic couplings (in Hz).
inter.coordinates	[nspins × 1] cell array of [1 × 3] row vectors	Cartesian coordinates of every spin (in Angstroms), used to determine point dipolar interactions. If a cell corresponding to a particular spin is left empty, that spin is assumed to not have any dipolar interactions with the rest of the system.

Examples:

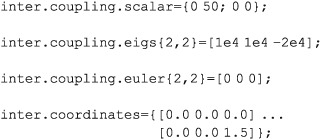

Spin–spin interactions may be specified in a variety of equivalent ways. The table below provides suggestions on specifying all common NMR interactions. *Spinach* supports most other types of magnetic resonance spectroscopy, but the corresponding interactions are outside the scope of this paper.

**Table:** 

Ways of specifying NMR interactions	
Nuclear chemical shift	Use inter.zeeman.scalar for isotropic chemical shifts, inter.zeeman.matrix for anisotropic chemical shift tensors supplied as matrices, or inter.zeeman.eigs & inter.zeeman.euler for anisotropic chemical shift tensors specified as eigenvalues and Euler angles.
Inter‐nuclear *J*‐coupling	Use inter.coupling.scalar; couplings that are specified multiple times, for example, between spins 1 and 2, and then again between spins 2 and 1, will be added together.
Inter‐nuclear dipolar coupling	Use inter.coordinates if nuclear coordinates are known (they will be converted into a dipolar interaction matrix internally), or inter.coupling.matrix for dipolar coupling supplied as a matrix, or inter.coupling.eigs & inter.coupling.euler for dipolar interactions supplied as eigenvalues and Euler angles.
Nuclear quadrupolar coupling	Best specified as an “interaction” of the nucleus with itself. Use inter.coupling.matrix or inter.coupling.eigs & inter.coupling.euler for quadrupolar interactions specified as eigenvalues and Euler angles.

A word of caution is in order about rotations in general and Euler angles in particular: there is no other subject in magnetic resonance that appears as innocent, and is actually as deadly, as three‐dimensional rotations. Space agencies have lost a few satellites to Euler angles, and every magnetic resonance theorist has gained a few grey hairs. Always store and publish your interactions as 3 × 3 matrices in Hz or ppm. *Spinach* would help you translate historical conventions — see the *Kernel Utilities* section of the online manual.

For partially oriented systems, the order matrix may be supplied to enable the simulation of orientation residuals of anisotropic interactions, for example,



inter.order_matrix=diag([1e−3 2e−3 −3e−3]);





Magnetic interaction parameters and atomic coordinates may also be imported directly into sys and inter data structures from *Gaussian* 16 and ORCA^17^ logs. In both cases, the log is first parsed and then the parse data are imported into *Spinach*, for example,



% Parse a Gaussian calculation log

props=gparse('../standard_systems/alanine.log');



% Import data into Spinach

[sys,inter]=g2spinach(props,{{'C','13C'},{'N','15N'}},[182.1 264.5],[]);



Further details on the parameters and options for the parser and the import functions are given in the manual. Spin system information may also be read from the spinsys{} field of SIMPSON^14^ *.in files.

Protein spin system composition and interaction information may be loaded from a pair of protein database files — a PDB file with atomic coordinates and a BMRB file with chemical shifts. The following call, used in the protein example set supplied with *Spinach*



**Figure 2:**
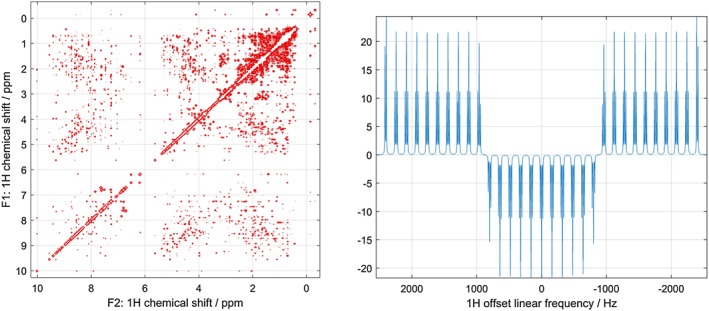
Left: fully quantum mechanical time‐domain Liouville‐space simulation of ubiquitin NOESY spectrum using full Redfield relaxation superoperator, performed as described in Edwards et al.^5^ Right: the result of a smoothed chirp inversion pulse on a 31‐spin system with strong nearest‐neighbour *J*‐couplings, followed by a homospoil gradient and a hard 90‐degree pulse. Both calculations are included into the standard example set supplied with *Spinach*

% Protein data import

options.pdb_mol=1;

options.select='all';

options.noshift='delete';

[sys,inter]=protein('1D3Z.pdb','1D3Z.bmrb',options);



would automatically create the necessary data structures, estimate all *J*‐couplings and some backbone chemical shift anisotropy (CSA) tensors. The detailed syntax description may be found in the manual. Nucleic acid data may be imported in a similar way:



% Import RNA data

options.noshift='delete';

[sys,inter]=nuclacid('example.pdb','example.txt',options);



*Spinach* example set contains several examples of protein and nucleic acid NMR simulations; some of the outputs of those calculations are shown in Figure 2. Further details may be found in our recent papers.^5^, ^6^, ^7^, ^18^

NMR calculations on ubiquitin‐size spin systems require 32 GB of RAM for the calculations that do not involve Redfield relaxation superoperator (such as HSQC and HNCOCA), and 128 GB of RAM for the calculations (NOESY and NOESY‐HSQC) that do.^5^ From about 100 spins onwards, the asymptotic scaling of both RAM requirements and CPU time with the size of the spin system in liquid state NMR simulations is linear.

## 4 RELAXATION AND CHEMICAL KINETICS

*Spinach* supports a large variety of relaxation theories, the most commonly used ones being *T*_1_/*T*_2_ approximation and Bloch–Redfield–Wangsness theory.^19^, ^20^, ^21^ The former simply assigns relaxation times to each spin in the system, and the latter assumes rotational diffusion and obtains relaxation rates from the interactions present in the system and the parameters of the diffusion process. Particulars of other relaxation theories may be found in the online documentation. Relaxation theory module in *Spinach* is uniquely powerful; it is implemented using very numerically efficient methods that can handle relaxation superoperators with dimension in excess of a million.^18^, ^22^

*Spinach* relaxation theory specification is a cell array listing all active relaxation theories, for example,



inter.relaxation={'redfield','t1_t2'};



requests both Redfield theory and *T*_1_/*T*_2_ theory. Within the *T*_1_/*T*_2_ theory, longitudinal and transverse relaxation rates in Hz should be provided for each spin. For example, in a three‐spin system:



inter.relaxation={'t1_t2'};

inter.r1_rates=[1.0 2.0 5.0];

inter.r2_rates=[5.0 7.0 9.0];



This would make all longitudinal states of each spin relax with rates *R*_1_ and all transverse states of each spin with rates *R*_2_. Strictly speaking, the *T*_1_/*T*_2_ relaxation model makes no mention of what happens to multi‐spin orders. *Spinach* therefore takes the liberty of making multi‐spin orders relax at the sum of the relaxation rates of their constituent operators. This is a reasonable approximation in most cases.

In order to use Redfield theory, the user must supply anisotropic parts for all relevant interactions, as well as one, two, or three rotational correlation times for each chemical species present in the system. The call with one rotational correlation time, for example,



inter.tau_c={1e−9};





would make *Spinach* assume isotropic rotational diffusion of what would be assumed to be a spherical molecule. A call with two rotational correlation times, for example,



inter.tau_c={[1e−9 2e−9]};



corresponds to axial rotational diffusion of what would be assumed to be an axially symmetric ellipsoid. The two‐element vector above gives the rotational correlation time around the symmetry axis of the ellipsoid (first element) and around an axis perpendicular to the symmetry axis (second element). The Z axis of the reference frame used to specify the interactions at the spin system specification stage must coincide with the symmetry axis of the rotational diffusion tensor. A call with three parameters, for example,



inter.tau_c={[1e−9 2e−9 3e−9]};



is assumed to give the three rotational correlation times of an arbitrary ellipsoid around X, Y, and Z principal axes (in that order) of its rotational diffusion tensor. The reference frame used to specify the interactions at the spin system specification stage must coincide with the eigenframe of the diffusion tensor.

**Figure 3:**
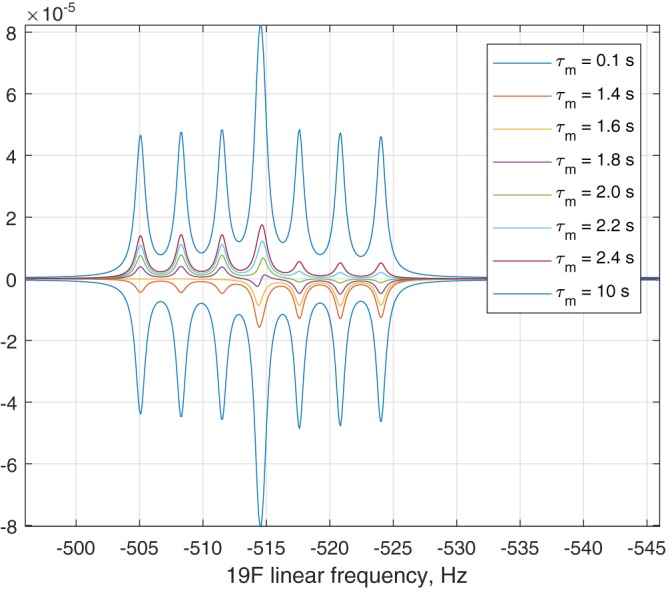
Inversion‐recovery ^19^F NMR spectrum of 1‐fluoro‐2,4‐dinitrobenzene as a function of mixing time, showing the effect of DD–CSA cross‐correlation described in detail by Grace and Kumar.^36^ The calculation is included into the standard example set supplied with *Spinach*

**Figure 4:**
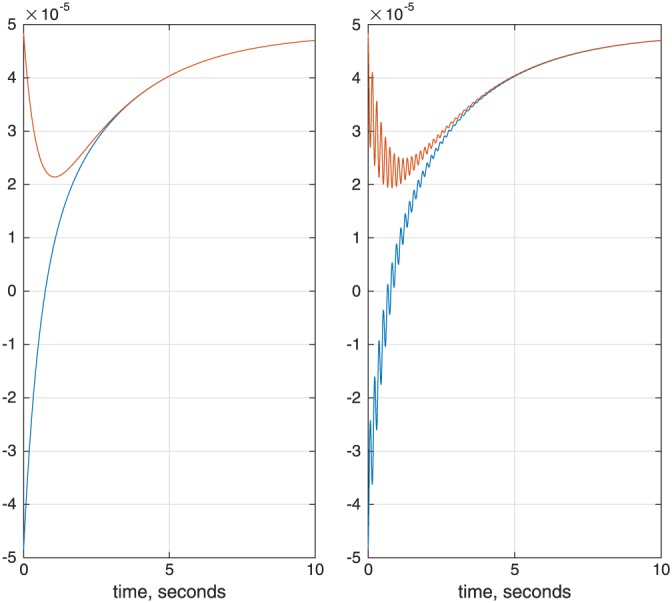
Longitudinal magnetisation as a function of time in a two‐proton spin system undergoing dipolar cross‐relaxation. The two spins are placed 2.0 Å apart, the rotational correlation time is set to 1.0 ns, the temperature is set to 298 K, the chemical shift difference is 0.01 ppm; the magnet field is 14.1 Tesla. One of the spins is inverted at time zero. Left: no *J*‐coupling between the spins. Right: zero‐quantum beats resulting from a 3.0 Hz *J*‐coupling. The calculations are included into the standard example set supplied with *Spinach*

The state to which the relaxation superoperator should be driving the system must be specified by setting the inter.equilibrium parameter. It controls the “thermalization” of the relaxation superoperator — a numerical correction that makes it drive the spin system to some prescribed thermal equilibrium state. The value of 'zero' causes the system to relax to the all‐zero state; specifying 'levante' or 'dibari' makes use of Levante–Ernst^23^ and DiBari–Levitt^24^ equilibrium correction methods, respectively. In that case, the spin temperature in the equilibrium state should also be specified, for example,



inter.temperature=298;



Not specifying a temperature makes the program use the high‐temperature approximation. Examples of relaxation theory simulations (available in the standard example set) are given in Figures 3 and 4.

Spinach has a very general chemical kinetics module that can handle arbitrary reaction networks, the only restriction being that the corresponding differential equations must be linear and must have the following general form:

(3) $$\frac{d}{\mathrm{dt}}\left(\begin{array}{c} \left[A\right]\\ \left[B\right]\\ \left[C\right]\\ \vdots \end{array}\right)=K\left(\begin{array}{c} \left[A\right]\\ \left[B\right]\\ \left[C\right]\\ \vdots \end{array}\right),$$

where **K** is the reaction rate matrix. For example,

(4) $$\begin{array}{c} A\;\overset{k_{1+}}{\to }B\overset{k_{2-}}{\underset{k_{2+}}{⇆}}C\\ \frac{d}{\mathrm{dt}}\left(\begin{array}{c} \;\;\left[\;\;A\;\;\right]\;\;\\ \;\;\left[\;\;B\;\;\right]\;\;\\ \;\;\left[\;\;C\;\;\right]\;\; \end{array}\right)=\left(\begin{array}{ccc} -k_{1+} & 0 & 0\\ k_{1+} & -k_{2+} & k_{2-}\\ 0 & k_{2+} & -k_{2-} \end{array}\right)\left(\begin{array}{c} \;\;\left[\;\;A\;\;\right]\;\;\\ \;\;\left[\;\;B\;\;\right]\;\;\\ \;\;\left[\;\;C\;\;\right]\;\; \end{array}\right) \end{array}.$$

*Spinach* expects the user to supply this matrix and the initial concentrations of the molecules. All of the molecules should be specified in the same input (simply listing their spins one after the other), and then *Spinach* should be told which spins belong to which molecule using inter.chem.parts variable, for example,



% Isotopes

sys.isotopes={'1H','13C','15N','1H','13C','15N'};



% Chemical shifts

inter.zeeman.scalar={1.0, 20.0, 15.0, 1.5, 25.0, 16.0};



% Spins 1,2,3 are molecule A; spins 4,5,6 are molecule B

inter.chem.parts={[1 2 3],[4 5 6]};



% Kinetic rate matrix (Hz)

inter.chem.rates=[−0.1 0.2; 0.1 −0.2];



% Initial concentrations (arbitrary units)

inter.chem.concs=[1.0, 2.0];



In the general case, the parameters, supplied at the spin system specification stage, must be

**Table:** 

Variable name	Variable type	Content
inter.chem.parts	A cell array of vectors containing integers	Individual vectors in the cell array must contain the numbers of spins that belong to each of the molecules in the chemical reaction, for example, {[1 2], [3 4]} indicates that spins 1 and 2 belong to the first molecule and spins 3 and 4 belong to the second molecule.
inter.chem.rates	A matrix of real numbers	Chemical reaction rate matrix between the molecules identified in inter.chem.parts variable.
inter.chem.concs	A vector of non‐negative real numbers	Initial concentrations of the molecules identified in inter.chem.parts variable.

The systems on either side of the reaction arrow must have the same number of spins, must have those spins specified in the same order, and must have the same basis set. Within Bloch–Redfield–Wangsness relaxation theory, different chemical compartments can have different rotational correlation times.

## 5 FORMALISM AND BASIS SET SPECIFICATION

*Spinach* supports three simulation formalisms: the standard |*α*⟩ and |*β*⟩ Zeeman basis used in most textbooks (colloquially known as “the Hilbert space”), the adjoint representation of the same (known as “the Liouville space”^25^), and a particularly convenient version of Liouville space that uses irreducible spherical tensor operators as the basis set.^4^ The formalism is chosen using bas.formalism parameter, for example,



bas.formalism='sphten−liouv';

**Table:** 

Formalism keyword	Formalism description
'sphten−liouv'	Liouville space formalism: the fundamental operators from which the basis set is built are single‐spin irreducible spherical tensors. These operators are ordered with respect to many common transformations and conservation laws encountered in magnetic resonance. Many operations may therefore be performed semi‐analytically. A lot of *Spinach* functionality either requires this formalism or operates most efficiently within it.
'zeeman−liouv'	Liouville space formalism: the fundamental operators from which the basis set is built are single transition operators between the projection states onto the Z axis. The state vector coefficients in this formalism are easy to interpret because they correspond to populations of standard textbook spin states. This formalism is essentially a vectorisation of 'zeeman−hilb'; it permits only limited state space reduction; most calculations would have exponential complexity scaling if this option is chosen.
'zeeman−hilb'	Hilbert space formalism: the fundamental states from which the basis set is built are the projection states onto the Z axis. This is the standard density operator formalism described in most magnetic resonance textbooks. Only the core functionality (operators, states, propagators, and evolution) is available. This option is mostly useful for backwards compatibility checks; it cannot support complicated relaxation theories or chemical kinetics. All calculations within this formalism would have exponential complexity scaling.

Basis sets are a highly technical topic — this tutorial specifically aims to avoid complicated mathematics. It would suffice to say that 'zeeman−hilb' is essentially the textbook route with Pauli matrices,^1^, ^2^, ^3^ and 'sphten−liouv' is its modern and very numerically efficient replacement.^5^, ^6^, ^26^, ^27^ The fastest algorithms that use incomplete basis sets^6^, ^7^ and have polynomial complexity scaling are only available within 'sphten−liouv' formalism. If the system has more than 20 spins, 'sphten−liouv' is the only realistic choice.^5^

The concept of an *incomplete* basis set is relatively new in magnetic resonance simulations,^6^ and an extended explanation is perhaps warranted. Every quantum state of the spin system may be described by a density matrix, and any matrix may be written as a linear combination of some basis matrices. In the simple case of one spin,

(5) $$\hat{\rho }=a{\hat{\sigma }}_{X}+b{\hat{\sigma }}_{Y}+c{\hat{\sigma }}_{Z},$$

where $\left\{{\hat{\sigma }}_{X},{\hat{\sigma }}_{Y},{\hat{\sigma }}_{Z}\right\}$ are Pauli matrices and {*a*, *b*, *c*} are complex numbers. In this case, the Pauli matrices are the “basis set”, and the complex numbers are the “expansion coefficients”. Systems with multiple spins have many more operators in the basis set: not only single‐spin operators but also multispin operators (*e.g.* ${\hat{\sigma }}_{Z}^{\left(1\right)}⊗\;{\hat{\sigma }}_{Z}^{\left(2\right)}$) that describe correlated simultaneous dynamics of multiple spins. It is here that approximations can be made: many such states are not populated for a variety of reasons.^6^, ^7^, ^26^, ^27^ The smaller the basis set, the faster the calculation becomes — but a balance must be struck between calculation speed and accuracy.

To run an *exact* (*i.e.* complete basis set) calculation in any formalism, set



bas.approximation='none';



This option requests a complete basis set, which is only practical up to about 20 spins in Hilbert space and 10 spins in Liouville space. *Approximate* calculations are those that use an incomplete basis set. The user is expected to provide the information that *Spinach* would use to build the basis set. The following frequently encountered choices are provided with the kernel:

**Table:** 

Approximation	Approximation description	Parameters
'IK−0'	Includes all product states between up to (and including) bas.level spins located anywhere within the system. For example, setting bas.level=5 would generate the basis that contains all spin correlations that involve five spins or fewer. The location of those spins is not taken into account.	bas.level
'IK−1'	Includes all product states between up to (and including) bas.level directly coupled spins and up to bas.space_level between spins that are closer together than the proximity cut‐off radius. This basis starts from IK−0, but then also drops correlations between very remote spins — if a pair of spins is not coupled in any way, even the two‐spin order between them is not actually needed. Here, bas.level controls the maximum correlation order for spins connected by couplings, and bas.space_level controls the maximum correlation order for spins that are within the proximity cut‐off radius.	bas.level, bas.space_level
'IK−2'	Includes, for every spin, all correlations with all directly coupled spins and correlations with up to (and including) bas.space_level with spins that are closer together than the user‐specified proximity cut‐off. This basis is similar to IK−1, except the truncation level around each spin is automatically set to the number of its direct coupling neighbours. This basis set can be quite large, but it is also very accurate.	bas.space_level

**Figure 5:**
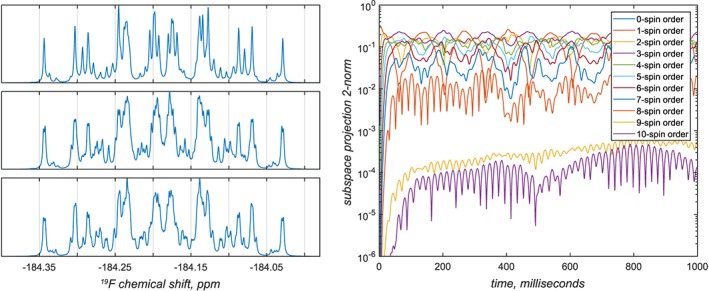
Convergence of the ^19^F NMR spectrum of anti‐3,4‐difluoro‐*n*‐heptane (16‐spin system) as a function of the basis truncation level. Top left: six‐spin orders and below. Middle left: seven‐spin orders and below. Bottom left: eight‐spin orders and below; this calculation is indistinguishable from the exact simulation to within about 10^−3^ in relative amplitude. Right: contributions from different orders of spin correlation to the system trajectory. The two traces in the lower part of the figure correspond to 9‐ and 10‐spin correlations — from their negligible magnitude, it is clear that for practical simulation purposes only correlations of up to eight spins need to be kept in the basis

The concept of a basis set in NMR simulations is illustrated in Figure 5. The spin system in question is anti‐3,4‐difluoro‐*n*‐heptane — with 16 spins, it is just outside of what is realistically possible to simulate with conventional time‐domain tools, even if symmetry and sparse matrix algebra is used. It is clear from the right panel of Figure 5 (note the logarithmic scale) that only correlations involving up to eight spins are populated to a significant extent in this system. This is a fundamentally important observation: the dimension of the full Liouville space in this system is in the billions, whereas the dimension of the reduced subspace is only 1,924,374; it is actually reduced further to 360,770 once the various symmetries and conservation laws are taken into account — *Spinach* does that automatically. It is instructive to go through the console log, which is reproduced below.

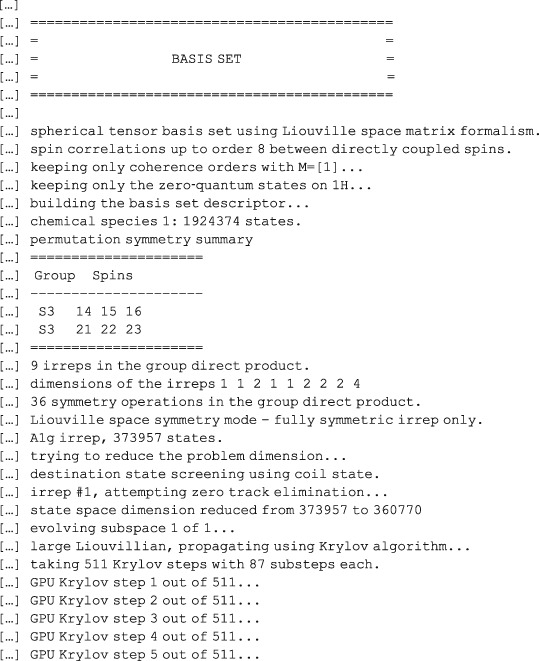

*Spinach* first applies the state space restriction to eight‐spin orders or less,^6^ then applies the conservation law with respect to the coherence order (+1 only in pulse‐acquire simulations with an ideal 90‐degree pulse), then applies the conservation law with respect to the observer spins (only zero‐quantum states are expected on protons), then applies the symmetry factorisation for the two methyl groups,^26^ then runs the zero track elimination,^7^ and finally engages the *Tesla K40* GPU that it has found in the system to push the density matrix through its time evolution using the Krylov algorithm.^7^ The whole calculation takes a few minutes. This ability to reduce matrix dimensions on the fly is the strongest side of *Spinach*.

The simulations producing Figure 5 are included into the standard example set supplied with versions 1.10 and later of *Spinach*; more technical information on the basis set specification may be found in the online manual and in the papers cited above — this practical tutorial is not the place for eye‐popping mathematics and computer science. For the purposes of getting started, the advice is quite simple: increase the basis set until the answer stops changing. In most liquid state NMR simulations, IK−2 with bas.space_level=3 and a 5 Å proximity cut‐off is sufficient. It is also possible to specify a completely custom basis set — see the online manual for further details. A technical discussion of the accuracy considerations when using incomplete basis sets is given in Karabanov et al.^28^

## 6 BUILT‐IN PULSE SEQUENCES

*Spinach* is designed to be extensible — our users write their own pulse sequences — but the following standard liquid state NMR experiments have been implemented by the *Spinach* team or donated by the users over the years: pulse‐acquire, inversion‐recovery, saturation‐recovery, CLIP‐HSQC, COSY, DQF‐COSY, HETCOR, HMQC, HNCO, HNCOCA, HSQC, LCOSY, NOESY, ROESY, TOCSY, and NOESY‐HSQC. The sequences rely on a common syntax that should be used to provide the relevant parameters, for example (HSQC):

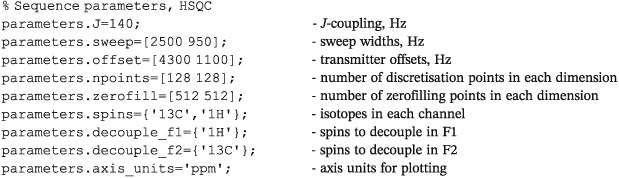

The list of necessary parameters is given in the documentation page for each pulse sequence. The responsibility for processing the free induction decay rests with the user. It may either be processed in *Matlab* (*Spinach* provides 1D, 2D, and 3D plotting functionality) or exported into a third‐party NMR processing package using *Matlab's* built‐in ASCII export functionality.

## 7 WRITING CUSTOM PULSE SEQUENCES

Writing *Spinach* simulations of pulse sequences is easier than writing them for NMR spectrometers because the syntax is sensible (here the instrument manufacturers get a dirty look) and phase cycles are not a problem — coherence selection may be performed by simply zeroing unwanted coherences.^26^ The next page shows the complete source code of the current *Spinach* implementation of the NOESY sequence^29^ that simulates anything from aziridine^30^ to ubiquitin,^5^ and also supports chemical kinetics. It is instructive to go through the code line by line.

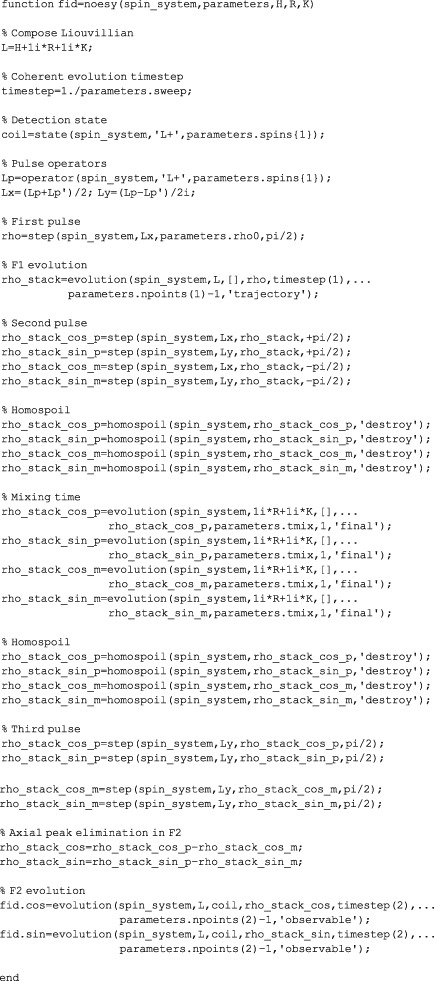

The pulse sequence does not need to worry about either the spin system or any relaxation/kinetics parameters: the corresponding operator or superoperator matrices (H for the Hamiltonian, R for the relaxation superoperator, and K for the kinetics superoperator) will simply be received from *Spinach* kernel — hence the argument list in the very first line. The next line puts all three operators together; their sum is called the Liouvillian and denoted L.

The next line deals with the evolution time step, which is inversely related to the sweep width that the user has specified in the parameters structure as illustrated in Section 6. The sequence then asks *Spinach* for the detection state ( ${\hat{L}}_{+}$ on all spins specified by the user) and the pulse operators ( ${\hat{L}}_{X}$ and ${\hat{L}}_{Y}$).

The sequence then performs the first pulse by taking the initial condition (supplied by the user in parameters.rho0) and using the step function. That function uses Krylov propagation^7^, ^31^ and is optimised for one‐off evolution events. The particulars are rather technical — *Spinach* manual contains further information.

The evolution command in the next line refers to the indirect dimension evolution. The arguments are the Liouvillian (L), the starting state (rho), the length of the time step, and the number of steps. Because this evolution period is incremented during the experiment, it makes sense to only run it once and to keep the entire trajectory — this is the meaning of the last argument in the function call. The trajectory is returned as a stack of state vectors (rho_stack), that is, a matrix made of individual column vectors arranged in the order of time from left to right.

At this point, the simulation splits into four independent batches: the next pulse is applied with four phases to create the components of the States quadrature^32^ and to eliminate the axial peaks in the F2 dimension that result from partial relaxation of the longitudinal magnetisation during the pulse sequence. A homospoil gradient is then applied to all four stacks (any states other than ${\hat{L}}_{Z}$ are simply zeroed out analytically).

The system is then sent through the mixing time using the evolution.m function provided by *Spinach* kernel. Its inner workings are complicated,^7^ but the user only needs to provide the evolution operator (relaxation and kinetics are needed here) and the duration of the evolution period. The mixing time is followed by another homospoil gradient and another pulse, with the same phase on all four batches. Axial peaks are then eliminated by subtracting the simulation pairs that differed in the direction of the second pulse.

Finally, the direct dimension evolution is run, and the magnetisation is detected on the coil state. The two components of the States quadrature are returned to the user.

Pulse sequences live in the experiments folder of *Spinach* distribution. All of them are extensively documented and also contain subroutines (called “grumblers”) that run detailed checks on the parameters supplied (or not supplied, as the case may be) by the user. Following that style is a good idea.

## 8 FITTING EXPERIMENTAL DATA

**Figure 6:**
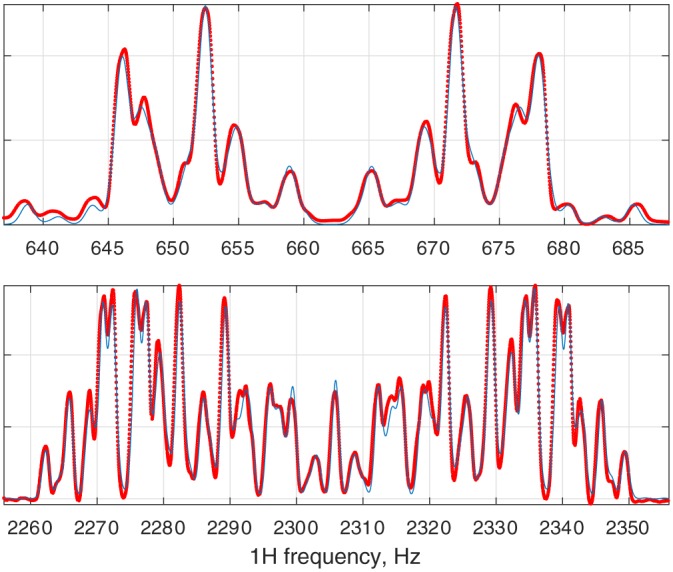
The result of the fitting of a 500 MHz ^1^H NMR spectrum of anti‐2,3‐difluoro‐*n*‐butane with respect to chemical shifts, *J*‐couplings, and line width. Red dots: experimental data. Blue lines: fitted spectrum. This calculation is included into the standard example set supplied with *Spinach*

Once a simulation is set up, converting it into a fitting procedure is quite easy — *Matlab* provides the necessary infrastructure. The only technicality is matching the X axis: point position and spacing in the simulation are not necessarily the same as in the experimental data. The experimental spectrum and the simulated one must therefore be interpolated into a common X axis point grid, for example,



sim_spec=interp1(sim_axis,sim_spec,exp_axis,'pchip');



This is a call to *Matlab's* built‐in 1D interpolation function that tells the program to take the data set with the ppm values for each point in the simulated spectrum listed in sim_axis, and values in sim_spec, and calculate the values of that spectrum at the points specified in exp_axis (the X axis of the experimental spectrum). The last option specifies a particular interpolation method — technical details may be found in *Matlab* documentation. Once the simulated and the experimental spectrum have the same X axis, they may be subtracted and the least squares error may be computed:



err=norm(real(expt_data)−real(sim_spec))^2;



This error is then minimised by *Matlab* as a function of relevant simulation parameters — multiple examples of such fitting runs are given in the standard example set supplied with *Spinach* (Figure 6).

Of the many error minimisation algorithms available in *Matlab*, Nelder–Mead simplex is recommended for situations when the initial guess is not particularly good,^33^ and LBFGS method for the refinement runs.^34^ Note that NMR fitting is a difficult problem — every parameter combination that makes any two lines overlap between the theoretical and the experimental spectrum is a local minimum on the error surface.

## 9 CASE STUDY 1—COSY45 OF ROTENONE

**Figure 7:**
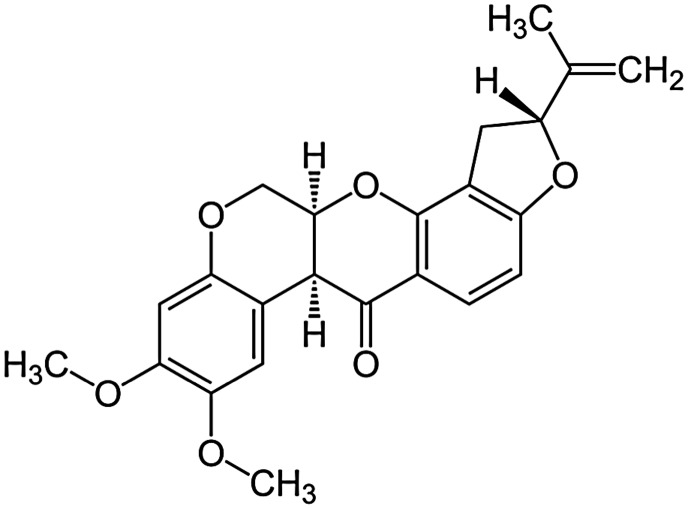
Chemical structure of rotenone

As a simple example that is both sufficiently easy to get started and sufficiently difficult to require *Spinach*, consider the simulation of a magnitude‐mode COSY45 spectrum of rotenone — a system with 22 spins and an irregular coupling pattern (Figure 7). This example is available in the example set supplied with *Spinach*.

The first thing *Spinach* requires is the function declaration:



function cosy45_rotenone()



This is not strictly necessary, but a good practice because this guarantees that *Matlab* starts the simulation with a clean background where no previously assigned variables exist. The second stage is to specify the isotopes, 22 protons in this case:

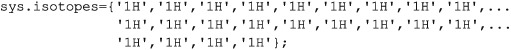

See the spin system specification section of the online manual for technical details on how to specify more complicated spin systems. The next step is to specify the magnet field (in Tesla):



sys.magnet=5.9;



then chemical shifts, in ppm for all protons:

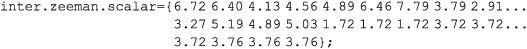

then all scalar couplings, in Hz:



inter.coupling.scalar{3,4}=12.1;

inter.coupling.scalar{4,5}=3.1;

inter.coupling.scalar{3,5}=1.0;

inter.coupling.scalar{3,8}=1.0;

inter.coupling.scalar{1,8}=1.0;

inter.coupling.scalar{6,7}=8.6;

inter.coupling.scalar{5,8}=4.1;

inter.coupling.scalar{7,9}=0.7;

inter.coupling.scalar{7,10}=0.7;

inter.coupling.scalar{9,10}=15.8;

inter.coupling.scalar{10,11}=9.8;

inter.coupling.scalar{9,11}=8.1;

inter.coupling.scalar{13,14}=1.5;

inter.coupling.scalar{12,14}=0.9;

inter.coupling.scalar{22,22}=0;



where the last line is necessary to tell *Matlab* that the array is 22 by 22 and all other elements are empty or zero.

The next stage is basis set specification. The complete basis set for a 22‐spin system is too large, and we must therefore rely on the restricted state space approximation (see the basis set specification section of the online manual and our recent papers^5^, ^6^, ^7^, ^26^, ^27^ for technical details of the basis set selection process). Here, we will be using the IK−2 basis with Liouville space formalism and no spatial proximity analysis because atomic coordinates are not supplied:



bas.formalism='sphten−liouv';

bas.approximation='IK−2';

bas.space_level=1;

bas.connectivity='scalar_couplings';



The three methyl groups contain magnetically equivalent protons, and this symmetry may optionally be used to reduce the calculation time:



bas.sym_group={'S3','S3','S3'};

bas.sym_spins={[14 15 16],[17 18 19],[20 21 22]};



This completes the basis set specification. The next stage is to specify the pulse sequence parameters. The full list of the parameters that *Spinach* stock pulse sequences require is given in the manual page for each sequence. The specific parameters required by the COSY sequence in this case are:



parameters.angle=pi/4;

parameters.offset=1200;

parameters.sweep=2000;

parameters.npoints=[512 512];

parameters.zerofill=[2048 2048];

parameters.spins={'1H'};

parameters.axis_units='ppm';



where the field names are intended to be self‐explanatory. This completes the specification of the spin system, of the basis set, and of the experiment parameters. The next stage is to give all that information to *Spinach*. This is accomplished by running the two housekeeping functions:



spin_system=create(sys,inter);

spin_system=basis(spin_system,bas);



Both print copious output to the console. This output should always be inspected carefully because it might contain warning messages. The next stage is simulation, which is carried out in liquid state (hence the liquid context function) with the assumptions set to 'nmr', indicating common high‐field NMR spectroscopy:



fid=liquid(spin_system,@cosy,parameters,'nmr');



The simulation returns the two‐dimensional free induction decay that should undergo apodization (cosine bell in both dimensions is a good choice here):



fid=apodization(fid,'cosbell−2d');



and Fourier transform (fft2 performs a two‐dimensional transform and fftshift moves the zero frequency to the centre of the spectrum — *Matlab*'s default is to have it on the edge):



spec=fftshift(fft2(fid,parameters.zerofill(2),parameters.zerofill(1)));



Finally, the spectrum is plotted (the many parameters of the plotting function are explained in the online manual):



 plot_2d(spin_system,spec,parameters,20,[0.0025 0.05 0.0025 0.05],2,256,6,'positive');



The whole simulation should take less than a minute on any modern laptop (Figure 8). Note that *Matlab* auto‐starts the parallelisation engine when it runs for the first time, that stage only happens once per *Matlab* session.

**Figure 8:**
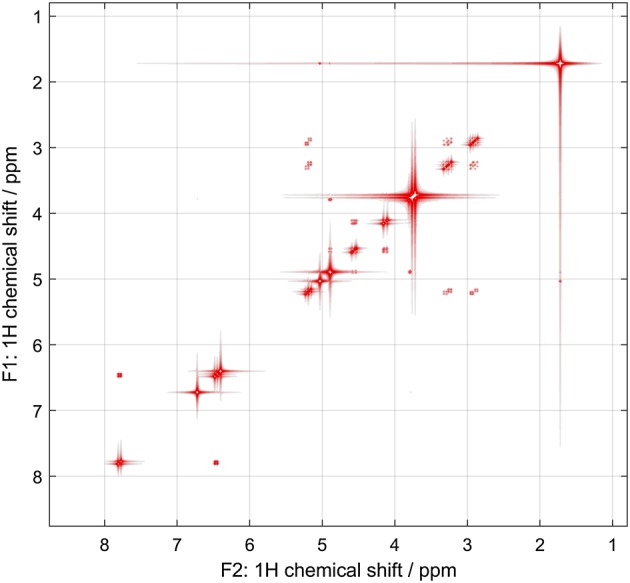
COSY‐45 simulation for rotenone, performed as described in the main text

## 10 CASE STUDY 2 — NOESY OF UBIQUITIN

This section describes the stages of setting up a simulation of a simple protein NMR experiment. Multiple examples of such simulations are available in the standard example set supplied with *Spinach*. You would need the following:

1. A suitably powerful computer. As a guidance, calculations that do not require a relaxation superoperator (HSQC and such) would need 16 GB of RAM to run ubiquitin, and the calculations that do need it (NOESY and such) would require 64 GB.
2. A PDB file containing Cartesian coordinates of every atom in the protein, including protons.
3. A BMRB file containing chemical shifts for those atoms that have been assigned. Unassigned atoms would either not appear in the simulation or end up with a chemical shift of −1 ppm (depending on the options specified, placing them at −1 ppm often helps with the subsequent assignment).

*Spinach* cross‐checks the amino acid sequence between the PDB and the BMRB file — any mismatch would produce an error message. Use the following command to import data and create *Spinach* input structures:



[sys,inter]=protein('pdb_file_name','bmrb_file_name',options);



The full list of options and the detailed descriptions of the subfields of sys and inter data structures are available in the manual. The protein import function above fills and returns the following fields:



sys.isotopes

sys.labels

inter.zeeman.scalar

inter.zeeman.matrix

inter.coupling.scalar

inter.coordinates



Field names are self‐explanatory: isotope names are placed into sys.isotopes, PDB labels of each atom are placed into sys.labels, chemical shifts are placed into inter.zeeman.scalar, rough guesses for nitrogen CSAs are placed into inter.zeeman.matrix (if you have accurate CSA tensors, you need to place them into the corresponding cells of inter.zeeman.matrix array after the import is complete), reasonable guesses of *J*‐couplings^5^ are placed into inter.coupling.scalar (if you have accurate *J*‐couplings, you would need to overwrite the values in inter.coupling.scalar after the import is complete); and PDB atom coordinates are placed into inter.coordinates; nothing else is imported or guessed. The detailed list of everything that happens when protein data are imported into *Spinach* is given in our recent paper^5^ and printed to the console at run time.

After the import is finished, the resulting sys and inter structures may be used by *Spinach*. Dipolar coupling tensors are computed automatically from atomic coordinates. Any additional information (quadrupolar coupling, unpaired electrons and associated interactions, *etc.*) can be added to sys and inter structures at this point.

At the next stage in the input preparation, you need to specify the magnet field and the cut‐off tolerances for the various interactions (which distances are “too large” for the dipolar coupling, and which *J*‐couplings are “too small” to be consequential). The top of the *Matlab* file should look similar to the following:



% Protein data

[sys,inter]=protein('1D3Z.pdb','1D3Z.bmrb',options);



% Magnet field

sys.magnet=21.1356;



% Tolerances

sys.tols.prox_cutoff=5.0;

sys.tols.inter_cutoff=2.0;



Cut‐off tolerance for proximity is specified in Angstrom and cut‐off for *J*‐coupling is specified in Hz. In the example above, dipolar couplings would not be taken into account between spins that are further than 5.0 Å apart and any *J*‐coupling smaller than 2.0 Hz would be ignored.

The next thing to be specified is the relaxation theory. Redfield relaxation theory is a very expensive option from the computational point of view — NOESY simulation for a 70‐residue protein requires about 64 GB of RAM (it was taking a terabyte in some of the early versions of *Spinach*). If you do not require accurate relaxation theory treatment, use something similar to the following:



% Relaxation theory

inter.relaxation='damp';

inter.damp_rate=5.0;



This requests a non‐selective damping at 5.0 Hz for all states (the relaxation superoperator would be a diagonal matrix with −5.0 on the diagonal). Alternatively, *Spinach* supports simple *T*_1_/*T*_2_ and Lindblad relaxation models — those are often sufficient; details are in the manual. However, if you do require accurate relaxation treatment (it is strictly necessary for NOESY spectra), the following should be supplied:



% Relaxation theory

inter.relaxation='redfield';

inter.rlx_keep='kite';

inter.tau_c=5e−9;



This requests full Redfield theory: DD, CSA, NQI, and all cross‐correlations thereof.^18^ Dipolar tensors are computed from atomic coordinates, CSAs and NQIs must be provided as described in Section 3. The middle line in the specification above requests the “Redfield kite” — cross‐relaxation is included between longitudinal states only. If you require the treatment of all cross‐relaxation processes, specify “secular” instead of “kite” — note that the simulation time would increase considerably. The last line specifies the rotational correlation time in seconds. It is important that you get this number right because all relaxation rates depend on it. *Spinach* relaxation module supports anisotropic rotational diffusion; further details are given in Section 3.

The next step is to choose a basis set. This is a very complicated topic (see the online manual), but the minimal basis set that produces quantitatively accurate results for proteins in liquid state is the following:



% Basis set

bas.formalism='sphten−liouv';

bas.approximation='IK−1';

bas.connectivity='scalar_couplings';

bas.level=5; bas.space_level=3;



This requests IK−1(5,3) connectivity‐adaptive basis set that includes local correlations of up to five spins on the *J*‐coupling graph and local correlations of up to three spins on the spatial proximity graph.^5^ In principle, some amino acid side chains (valine and isoleucine) require correlations of more than five spins to be present in the basis set to get their multiplicity absolutely right, but the multiplet structure of the corresponding signals is never actually resolved in protein NMR spectra. An absolutely bulletproof basis here would be IK−1(8,3), but in this case, it simply produces the same answer after a much longer calculation.

The next stage is to call *Spinach* constructor functions and generate the spin_system data structure that contains all information about the spin system and is required by most high‐level *Spinach* functions as the first argument:



% Create the spin system structure

spin_system=create(sys,inter);



% Kill carbons and nitrogens

spin_system=kill_spin(spin_system,strcmp('13C',spin_system.comp.isotopes));

spin_system=kill_spin(spin_system,strcmp('15N',spin_system.comp.isotopes));



% Build the basis

spin_system=basis(spin_system,bas);



The two lines in the middle are optional — in this case, they request the removal of all carbon and nitrogen spins from the spin system. This is necessary for the NOESY simulation but should not be done for HSQC, HNCO, and other sequences that require the presence of ^15^N and ^13^C spins.

The next stage is to specify experiment parameters. In the case of 2D NOESY, the following is a reasonable set:



% Sequence parameters

parameters.tmix=0.065;

parameters.offset=4250;

parameters.sweep=10750;

parameters.npoints=[512 512];

parameters.zerofill=[2048 2048];

parameters.spins={'1H'};

parameters.axis_units='ppm';

parameters.rho0=state(spin_system,'Lz','1H');



As the names of the parameters suggest, this requests a mixing time of 65 ms, frequency offset of 4250 Hz, sweep width of 10750 Hz, 512 points to be acquired in both dimensions, and zerofilled to 2048 points in both dimensions, the sequence is operating on ^1^H nuclei, axis units should be ppm, and the initial condition is ${\hat{L}}_{Z}$ on protons.

The next stage is the actual simulation. In the case of 2D NOESY, the syntax is



% Simulation

fid=liquid(spin_system,@noesy,parameters,'nmr');



The choice of the outer function reflects the fact that we are running a liquid state simulation (*Spinach* supports all other types of magnetic resonance spectroscopy and imaging), spin_system is the data structure containing the information about the system, noesy is the name of the pulse sequence we are running (@ symbol is a *Matlab* technicality — it denotes a function handle), and the various fields of the parameters argument have all been set above. The result is a 2D free induction decay that is ready for standard NMR data processing. Depending on the pulse sequence, it may be a simple array of complex numbers, or it might contain subfields, such as fid.cos and fid.sin, that are used in States quadrature processing of phase‐sensitive experiments.

The next stage is apodization, which may be accomplished using any of the window functions available in *Spinach* — the complete list is in the manual. In this particular case, we will use Gaussian apodization:



% Apodization

fid.cos=apodization(fid.cos,'gaussian‐2d',5);

fid.sin=apodization(fid.sin,'gaussian‐2d',5);



where the last argument is the decay rate (per data set point) of the Gaussian function — this parameter should be increased until the sinc wiggles disappear from the spectrum. Good practical advice on spectral apodization was published by Vosegaard and Nielsen.^35^

The next stage is Fourier transform and quadrature processing. For a 2D NOESY simulation, States quadrature processing is necessary:



% F2 Fourier transform

f1_cos=real(fftshift(fft(fid.cos,parameters.zerofill(2),1),1));

f1_sin=real(fftshift(fft(fid.sin,parameters.zerofill(2),1),1));



% States signal

f1_states=f1_cos−1i*f1_sin;



% F1 Fourier transform

spectrum=fftshift(fft(f1_states,parameters.zerofill(1),2),2);



This is standard *Matlab* Fourier transform syntax: fft is the command that performs the transform and fftshift performs a cyclic shift that moves the zero frequency to the centre of the spectrum. Finally, the plotting function produces a contour plot:



% Plotting

plot_2d(spin_system,−real(spectrum),parameters,20,...

[0.01 0.05 0.01 0.05],2,256,6,'positive');



2D and 3D plotting functions in Spinach have a significant number of adjustable parameters that are described in the manual. The last argument tells the plotter to ignore negative peaks. If those are expected in the spectrum, the argument should be 'both'. The output is shown in Figure 2.
